# Functional Reconstruction of Temporomandibular Joint after Resection of Pigmented Villonodular Synovitis with Extension to Infratemporal Fossa and Skull Base: A Case Report

**DOI:** 10.1055/s-0036-1587693

**Published:** 2016-08-10

**Authors:** Eduardo de Arnaldo Silva Vellutini, Nivaldo Alonso, Sérgio Samir Arap, Luís Felipe Silva Godoy, Ricardo Antenor de Souza e Souza, Rômulo Loss Mattedi, Matheus Fernandes de Oliveira

**Affiliations:** 1 Department of Neurosurgery, DFV Neuro, São Paulo, Brazil; 2 Department of Plastic Surgery, Faculdade de Medicina da Universidade de São Paulo, São Paulo, Brazil; 3 Department of Head and Neck Surgery, Faculdade de Medicina da Universidade de São Paulo, São Paulo, Brazil; 4 Radiology Service, Faculdade de Medicina da Universidade de São Paulo, São Paulo, Brazil; 5 Department of Pathology, Hospital Sírio Libanês, São Paulo, Brazil

**Keywords:** temporomandibular joint, skull base, synovitis, treatment

## Abstract

**Introduction** Pigmented villonodular synovitis (PVNS) is a benign but aggressive lesion arising from sinovia. The temporomandibular joint (TMJ) is hardly ever involved.

**Methods** We describe a case of PVNS arising in the left TMJ involving infratemporal fossa soft tissue and the skull base; we also present the reconstruction.

**Results** A 37-year-old woman had progressive mandibular swelling for 6 months. Computed tomography of the skull revealed an osteolytic lesion in the left TMJ, involving the upper mandible, condyle, and glenoid fossa and extending to the infratemporal fossa and fossa media through a defect in temporal bone. Surgical management included a left pterional craniotomy to reach the temporal skull base and resect the intracranial tumor and a facial approach with partial left mandibulectomy and resection of left condyle, glenoid fossa, and tumor removal in infratemporal fossa. Mandible function was restored with prosthetic reconstruction of the condyle. She progressively started to eat solid foods after 3 months, becoming increasingly functional and asymptomatic. At 30 months' follow-up, she had no sign of tumoral recurrence and showed asymptomatic and normal TMJ function.

**Conclusion** PVNS should be considered in the differential diagnosis of bone neoplasms affecting young patients. In such cases, radical excision is mandatory and TMJ prosthesis for local reconstruction may be used to preserve functionality.

Pigmented villonodular synovitis (PVNS) is a benign but aggressive proliferative lesion arising from sinovia, most commonly found in the joints of the long bones. The temporomandibular joint (TMJ) is hardly ever involved. 1 2 3 4

To date, 58 cases of PVNS have been described, and 19 of them presented with intracranial extension. In these cases, the ideal approach to lesion excision and functional reconstruction is not standardized, and several strategies may be applied. 2 5 6 7 8 9

We describe a case of PVNS arising in the left TMJ and involving infratemporal fossa soft tissue and the skull base; we also present the reconstruction with a prosthesis.

## Case Report

A 37-year-old woman had intermittent mild to moderate headache 7 years previously. Computed tomography (CT) was normal at that time. The main diagnosis was a primary headache, and she started to use analgesics.

Six months ago, she presented with dizziness and worsening of headache in the left side, especially in the temporal and mandibular regions. At that time, mandibular swelling was noted, and she presented with pain when masticating. Neurologic examination revealed no alterations. There was no previous history of trauma.

**Fig. 1:**
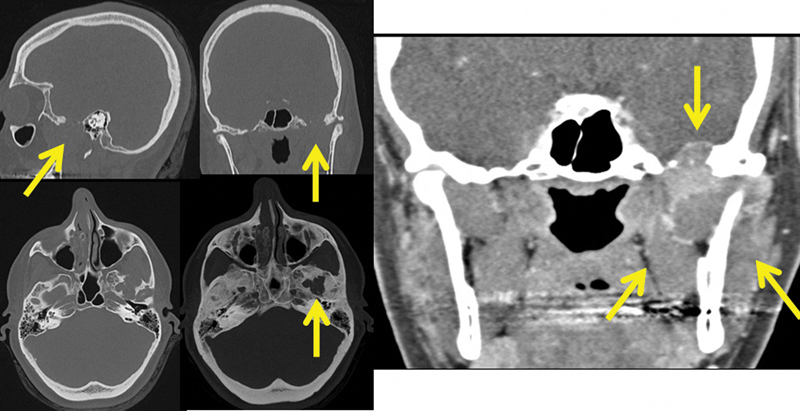
Computed tomography of the skull revealed an osteolytic lesion in left temporomandibular joint (arrows). Lesion had slight contrast enhancement.

**Fig. 2:**
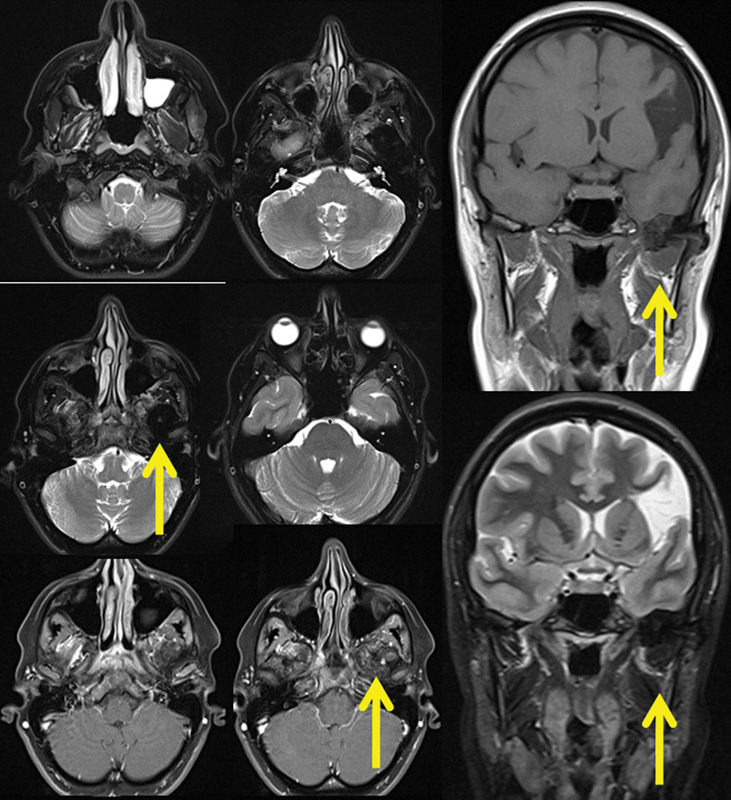
Magnetic resonance imaging of the skull identified a mass with low signal in T1- and T2-weighted images, with small focal enhancement with gadolinium.

CT of the skull revealed an osteolytic lesion in the left TMJ, involving the upper mandible, condyle, and glenoid fossa, compromising and extending to the soft tissue in the infratemporal fossa and invading the fossa media through a defect in the temporal bone. The lesion had slight contrast enhancement ( Fig. 1 ). Magnetic resonance imaging identified a mass with low signal in T1- and T2-weighted images, with small focal enhancement with gadolinium ( Fig. 2 ).

**Fig. 3:**
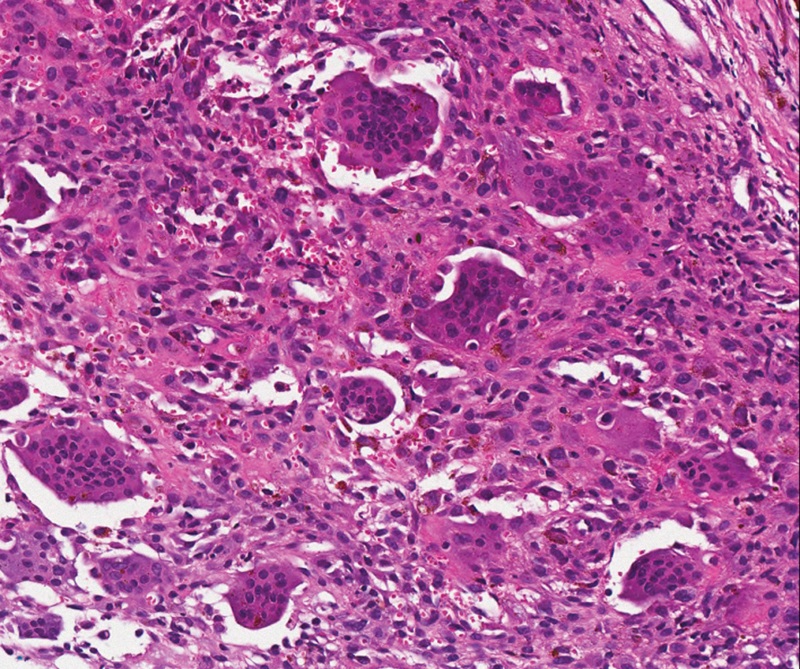
Hematoxylin-eosin pathologic image (×200). Several giant and multinucleated cells, with regular nuclei. Giant cell tumor compatible with pigmented villonodular synovitis.

A CT-guided percutaneous biopsy disclosed a mesenchymal neoplasm with several giant and multinucleated cells, with regular nuclei, similar to stromal cells, and new and old foci of hemorrhage. The pathologic report confirmed a giant cell tumor compatible with PVNS ( Fig. 3 ).

**Fig. 4:**
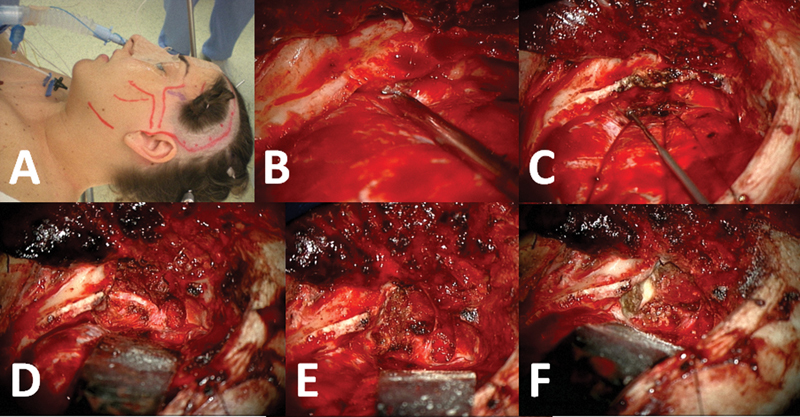
Cranial approach with a left pterional craniotomy. (A) Positioning in head holder, skin marking, and nasotracheal intubation. (B) Just after performing pterional craniotomy. (C) Dissection of temporal base and visualization of lesion. (D) Basal temporal osteotomy, revealing temporomandibular joint (TMJ). (E) Removal of TMJ and tumoral resection. (F) After tumoral resection.

Treatment included surgical removal with free margin resection. The surgical management involved multidisciplinary principles. A left pterional craniotomy was performed to reach the temporal skull base, resect intracranial tumors, and debride the skull base. The operative field was entirely extradural; the tumor was focused in bone, and no dural involvement was noticed ( Fig. 4 ). The tumor extended from the glenoidal cavity in the lateral aspect to the external margin of the foramen rotundum in the medial aspect, sparing the trigeminal branches. Surgical dissection respected these margins, allowing tumor removal and preserving the cranial nerves.

Simultaneously, a facial approach was used with partial left mandibulectomy; the left condyle, glenoid fossa, and tumor in the infratemporal fossa were excised. The lesion was rigid and had little bleeding. Mandibular function was restored with a prosthetic reconstruction of the condyle.

**Fig. 5:**
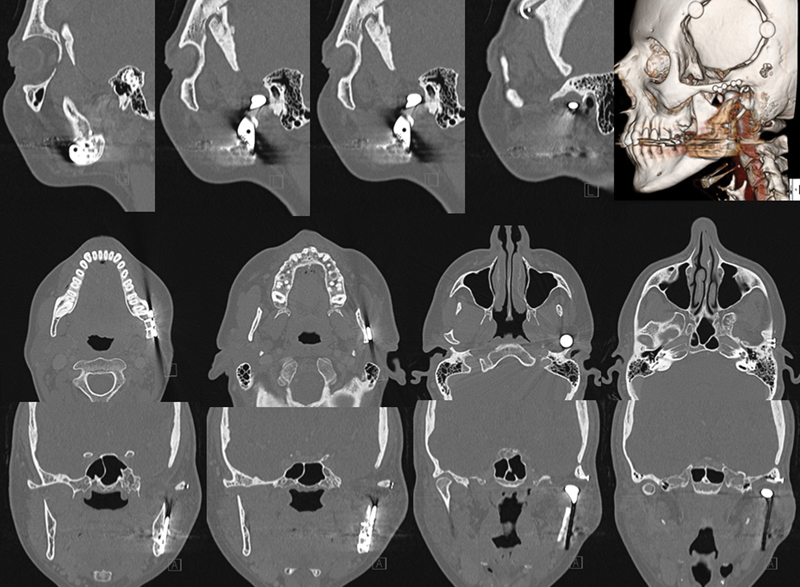
Follow-up image after 30 months, with no signs of tumoral recurrence.

After surgery, the patient was restricted to liquid foods. Neurologic examination revealed House-Brackmann grade III facial nerve paresis and hypesthesia in the left V2 territory. The patient has since reported to regular follow-up. At 20 days' follow-up, there was still grade II facial nerve paresis and V2 hypesthesia. She had progressed to a pasty diet. Three-month follow-up revealed mild TMJ pain, with resolution of facial paresis and hypesthesia. She progressively started to eat solid foods after 3 months, becoming increasingly functional and asymptomatic. At 30 months' follow-up, there was no sign of tumoral recurrence, and the patient had asymptomatic and normal TMJ function ( Fig. 5 ). No adjuvant therapy was then indicated.

## Discussion

PVNS is a rare and benign neoplasm with excellent outcomes, even with intracranial extension. 1 2 3 4

There are no clinical symptoms specific to PVNS. 1 2 3 4 Patients usually present with pain and a limited range of motion caused by proximity of the tumor to the related area. Patients may complain of pain behind the ear on the lesion side, hearing difficulty, swelling in the concerned region, and facial paralysis. 1 2 3 4

Few reports of PVNS involve the TMJ, and approximately one-third have invasion of the skull base. 1 2 3 4 5 6 Complete excision of the tumor is the established treatment of choice, and recurrence depends upon the adequacy of treatment. In cases with recurrence, additional surgery may be tried and radiotherapy is usually advocated. 1 2 3 4 5 6

Safaee et al described five patients who underwent surgical resection, with 1 recurrence at 61 months. 2 In the current literature, 58 patients with PVNS were reported, 19 of whom had intracranial involvement. Intracranial extension was not associated with an increased rate of recurrence. 2

However, when these lesions involved the TMJ, complete resection with free margins becomes technically difficult, and there are issues to be addressed concerning reconstruction of the TMJ. 7 8 9 Osteoarticular autograft, allograft, and prosthetic reconstruction have been described. 7 8 9 A previous report suggested that the use of autologous tissue enables the rapid incorporation with a lower risk of infection, especially with vascularized graft. 7 However, in cases with wide margin resection and need for reconstruction of glenoid fossa and TMJ, some complications may happen with the use of autologous grafts, including unpredictable bone integration and even graft resorption due to joint mobilityjoint. 8 9 On the other hand, fibrosis, scar tissue, and ankylosis may occur, limiting opening and closing of the mouth. 8 9

In our case, we diagnosed a PVNS in a young woman with TMJ pain and swelling. Image studies revealed a mass in the TMJ extending to the infratemporal fossa and skull base. A combined multidisciplinary approach was used to resect the lesion and allowed for the reconstruction of the TMJ. The facial nerve lesion was peripheral, especially in the mandibular branch, due to external traction during parotidectomy. The lesion was partial and temporary, with full recovery after 3 months, which was not related to the intracranial approach. Facial nerve dissection during parotidectomy was important to allow surgical reconstruction of the TMJ.

We performed a zygomatic osteotomy to allow an anterior approach to the glenoidal cavity and to the skull base. However, a zygomatic osteotomy was unnecessary because parotidectomy was done by an external approach to allow mandibular condyle resection and tumor removal.

The prosthesis was applied to reconstruct the glenoidal cavity after resection of the mandible condyle and to keep the height of the ascending mandibular ramus. Without the resected condyle, there would be mandibular asymmetry (static and dynamic) with consequent dental occlusion dysfunction, leading to masticatory and phonatory dysfunction.

Reconstruction of the glenoidal cavity was the most important step; there are other alternatives such as bone graft, but bone graft is not usually a long-lasting material. In young patients, the glenoidal cavity must be resistant to support pressure and action, which is why we used a prosthesis instead of bone graft. There was low morbidity and complete functional recovery after 3 months. The patient also returned to singing in the choir. Additionally, the patient was asymptomatic after 2 years, and there have been no signs of tumor recurrence in routine follow-up.

We highlight the differential diagnosis of bone neoplasms affecting young patients. In such cases, radical excision is mandatory and TMJ prosthetic for local reconstruction may preserve functionality.
